# Impact of Type and Degree of Defect on Selected Properties of Graphene Quantum Dots

**DOI:** 10.3390/molecules30173521

**Published:** 2025-08-28

**Authors:** Lukasz Kaczmarek, Piotr Zawadzki, Magdalena Balik, Piotr Kosobudzki, Adam Roslak

**Affiliations:** Institute of Materials Science and Engineering, Lodz University of Technology, Stefanowskiego 1/15 St., 90-537 Lodz, Poland; lukasz.kaczmarek@p.lodz.pl (L.K.); magdalena.balik@p.lodz.pl (M.B.); piotr.kosobudzki@dokt.p.lodz.pl (P.K.); adam.roslak@dokt.p.lodz.pl (A.R.)

**Keywords:** graphene quantum dots, size effect, photoluminescence, defects, simulation

## Abstract

Graphene quantum dots (QGDs), as nascent carbon-based materials, demonstrate remarkable promise in many different applications. Thanks to excellent electrical and thermal properties, great biocompatibility, feasibility of surface functionalization and low cytotoxicity, QGDs can be any material and have many applications, from elastic PV panels to drug delivery. This paper concentrates on relating the structure of the QGD (which is the result of the synthesis method used and consequently the variable degree of defect, the possible presence of functional groups especially in the defect region, etc.) to the resulting physicochemical properties. Therefore, the aim of this study is to theoretically relate and determine the effect of defect amount and type on the value of the HOMO–LUMO gap with respect to possible QGD luminescence colors. Finally, it presents a direction in new graphene-based materials synthesis, where every single defect has a huge impact on its properties.

## 1. Introduction

Currently, there are two groups of methods for the synthesis of graphene quantum dots: top–down (grinding carbon materials: graphite, graphene, carbon fibers, carbon nanotubes) and bottom–up (synthesis from carbon precursors). Regarding top–down methods, the most popular techniques include intercalation of graphite followed by exfoliation [1], electrochemical cutting [2], electrochemical oxidation [3], acidic exfoliation from precursors in the form of carbon fiber [4], carbon nanotube [5], and hydrothermal exfoliation of GO [6]. On the other hand, the main disadvantages of top–down methods include lack of control over the amount of QGD produced and their size dispersion. In addition, these methods are characterized by relatively low efficiency, reaching only 10% QGD with a given size distribution and a high degree of deterioration in graphene systems. Moreover, these methods often require the use of aggressive reaction environments (e.g., chemical oxidation method) or high pressures and temperatures (e.g., hydrothermal method). The most important disadvantage of the above methods is the difficulty in controlling the quality of the structure and the size of the obtained carbon structures. This fact makes it impossible to fractionate QGD in terms of the assessment of the physicochemical property characteristics for the given size ranges. Among all types of QGD synthesis, both in top–down and bottom–up methods, there are many problems related to correlating the reaction parameters with thermodynamic stability, as well as the physical and chemical properties of QGD [7,8]. This fact significantly limits their potential application in areas such as energy conversion (transparent photovoltaic cells with unprecedented efficiency over 35%) [9], energy storage systems [10], light-emitting diodes [11], cancer cell markers [12], optoelectronic systems [13]. However, the lack of control of their synthesis process in terms of size, degree of defect, as well as the proportion of hydrogen to carbon significantly limits their use.

For this reason, bottom–up techniques play an increasingly important role, in the case of which the following can be distinguished: synthesis from organic precursors, e.g., synthesis with the use of a precursor in the form of glucose in the NH_3_ environment [14], pyrolysis of glucose [15], vapor deposition (CVD) on a copper foil [16,17], uses of polymers, e.g., Poly(3-alkylthiophenes) (P3ATs) of different chain lengths (alkaneC12, alkaneC14, alkaneC16) [18]. The above methods, like the bottom–up group, also show difficulties in controling the size of the synthesized structures. On the other hand, the methods of QGD synthesis, which use simple sugars initiated with microwaves, are gaining more importance [19,20,21]. The use of fruit extracts with simultaneous control of the synthesis parameters (environment, range of reaction temperature changes, substrate concentration, microwave radiation power) may lead to the production of graphene systems with a very narrow size distribution, [Patent pending: PL440978].

However, irrespective of which synthesis methods are used, the structures of the produced QGDs will invariably be characterized by defects. The presence of defects has a direct impact on the value of the HOMO-LUMO energy levels. Theoretically the value of the HOMO–LUMO gap for an ideal graphene structure is 0. The reason is that there is a zero-energy gap between the base band and the conduction band. Every change in structure disrupts the electron distribution (presence of C atom vacancies, presence of C-H bonds on edges, additional functional groups in defects: edges or vacancies, etc.,) and thus changes the value of the HOMO–LUMO gap.

For QGDs, an increase in their surface area results in a decrease in the HOMO–LUMO gap and, conversely, it results from an enhancement in the orderliness of the molecular system expressed as an increase in the proportion of sp^2^/sp^3^, which is an elevation in the proportion of aromatic systems relative to potential defects. Moreover, for HOMO–LUMO gap values in the range 2.2 eV–3.1 eV, green or blue color emission is observed. Therefore, it is possible to imagine QGDs with significantly different structures resulting from, e.g., a different degree of defect or a different form factor, but with the same color emission in fluorescence. This fact causes many complications, especially if one wants the best possible electrical properties required for, e.g., transparent photovoltaic cells. In this context, the structure is crucial to form a layout with percolation properties enabling the transport of excited electrons. Every defect encountered significantly reduces electron transfer, thus limiting cell efficiency.

Consequently, the problem is related to the structure of the QGD (which is the result of the synthesis method used and consequently the variable degree of defect, the possible presence of functional groups especially in the defect region, etc.) to the resulting physicochemical properties. Therefore, the aim of this study is to theoretically relate and determine the effect of defect amount and type on the value of the HOMO–LUMO gap with respect to possible QGD luminescence colors.

## 2. Results and Discussion

### 2.1. Energy Variation of the System in Relation to the Type of QGD Defect

**Figure 1:**
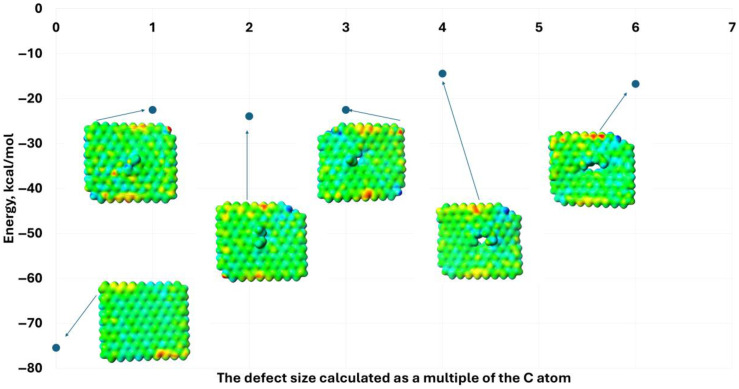
Values of the graphene quantum dot system energy as a function of the size of the carbon atom vacancy defect, along with examples of electron distributions for modeled QGDs (l/d = 2.47/2.13 nm), respectively: without defect and with vacancies up to 6 carbon atoms in size.

Initially, the analysis was conducted for a non-defective graphene quantum dot system composed of 162 carbon and 36 hydrogen atoms. This arrangement shows an energy of −75 kcal/mol after the process of the optimization. To determine the effect of structural rearrangement of graphene quantum dots on their system energy and electron distribution, as well as UV–Vis absorption spectra, the previously optimized structure was then made defective in the range of 1 to 6 carbon atoms, and the structure optimization procedure was carried out independently for each type of defect (Figure 1).

**Figure 2:**
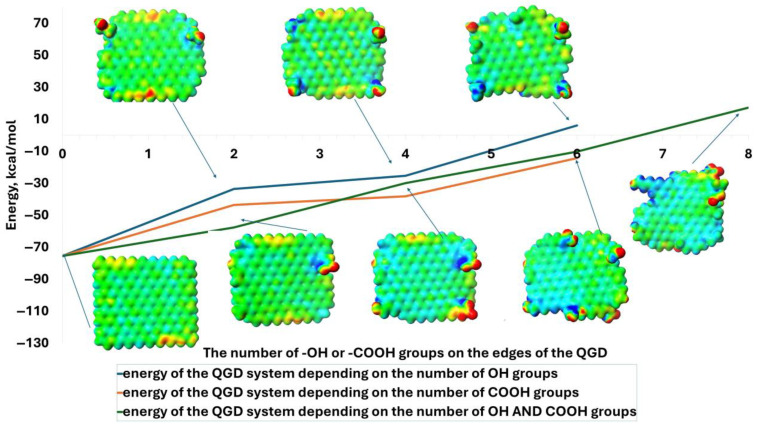
Energy value of the graphene quantum dot system depending on the type and number of -OH and -COOH functional groups, as well as the -OH and -COOH groups located at the edges, along with examples of electron distributions of modeled QGDs.

The defect of graphene quantum dots, interpreted as a multiple carbon vacancy, causes an increase in the energy of the system. The absence of just one carbon atom increases the energy of the system from approximately −75 kcal/mol to −22 kcal/mol (Figure 2). In contrast, an increase in the size of the analyzed defect, between 1 and 6 carbon atoms, has no further significant effect on the overall energy change in the system. In this case, the energy of the arrangement is around −20 kcal/mol. This is because defects in the QGD system disrupts the homogeneity of the electron distribution within it. Noticeably electron-poorer (zone of defect—carbon vacancies and edges—blue) and electron-richer (edge zones—red) regions are formed. The polarization of the layout, but also the flatness alteration of the QGD as a result of the hybridization change within the defect from sp^2^ to sp^3^, can have a significant impact on electron excitation and transport in percolation superstructures. Ultimately, this fact may translate into a reduction in, e.g., the photovoltaic effect, but may also affect the level of sensitivity of the electronics used to build the sensors.

**Figure 3:**
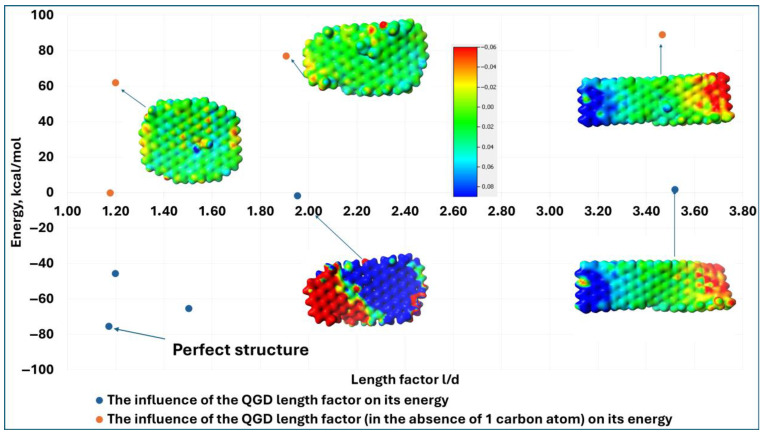
Energy value of the graphene quantum dot system depending on the value of the parameter l/d.

The increase in the energy of the system is also observed when the parameter l/d increases (Figure 3). For an ideal layout (the value of the parameter l/d is 1.17 and the corresponding system energy is 75 kcal/mol), a rise in the parameter l/d to 1.95 causes the system energy to grow to a value of 2 kcal/mol. A further increase in it does not significantly change the energy value of the layout. It proves that flawed systems, as well as those with an elongated structure, are not preferred for possible synthesis. As opposed to rounded structures, which are energetically preferred from the point of view of their relatively high thermodynamic stability, structurally disordered systems are not preferred from a thermodynamic perspective.

To ensure structural comparability, the initial pristine QGD model was benchmarked against literature-reported geometries [9]. Functional groups (-OH, -COOH) were added at symmetrical edge positions or within vacancies to minimize artificial polarization. The choice of defect sizes (1–6 C atoms) reflects realistic structural disruptions reported in experimentally obtained QGDs [18].

Although our work focuses on trends, the resulting HOMO–LUMO gaps (1.6–3.0 eV) and peak shifts in UV–Vis absorption spectra closely correspond to experimental data for QGDs of similar size and surface chemistry [1].

Furthermore, the simultaneous defects in layouts with variable parameter l/d by removing only one carbon atom results in a step gain in the energy of the system for l/d = 1.2 and for 1.95 with one carbon vacancy, the system energies are 0 kcal/mol and +78 kcal/mol, respectively. What is also confirmed by this fact is that, from a thermodynamic point of view, these systems are not preferable. In addition, the substitution of QGDs with -OH or -COOH groups, as well as making additional repairs to the structure, causes a change in their polarity. This may be of great importance during the deposition of QGDs on various substrates to create, for example, sensory systems or structures that serve as drug carriers, bactericidal systems, etc. The reason for this is that a transformation of the electron character will affect the physicochemical interactions with the surface (may increase or decrease adhesion to the substrate) and may also translate into the degree of interaction in a given area. This last example is extremely relevant to the generation of percolation systems that form the basis of transparent materials with photovoltaic properties. Mutually polarized dots can achieve superstructures that will attract each other while generating the conditions to create such spaces, allowing the excited electron to jump between dots. This effect will undoubtedly maximize the efficiency of silicon-free and transparent photovoltaic cells [22].

### 2.2. Variations in the UV–Vis Absorption Spectra Depending on the Defect Type of the QGD

**Figure 4:**
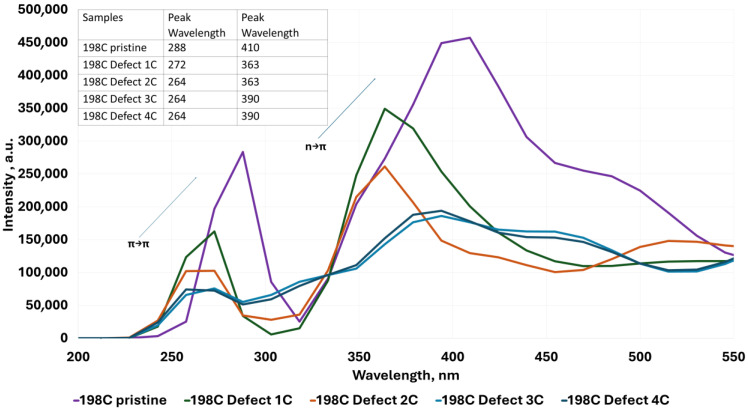
Examples of UV–Vis absorption spectra for the ideal modeled graphene quantum dot system and for defected systems of 1 to 4 C atoms, respectively.

**Figure 5:**
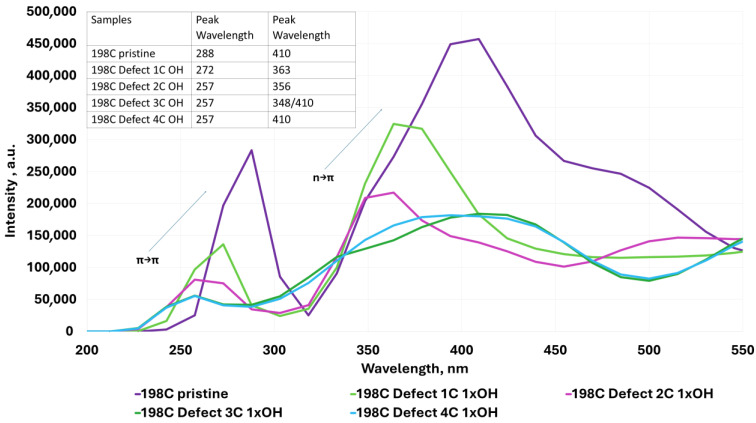
Examples of UV–Vis absorption spectra for the ideal modeled graphene quantum dot system and for defected systems with 1 C atom and -OH group substituted in the defect region.

**Figure 6:**
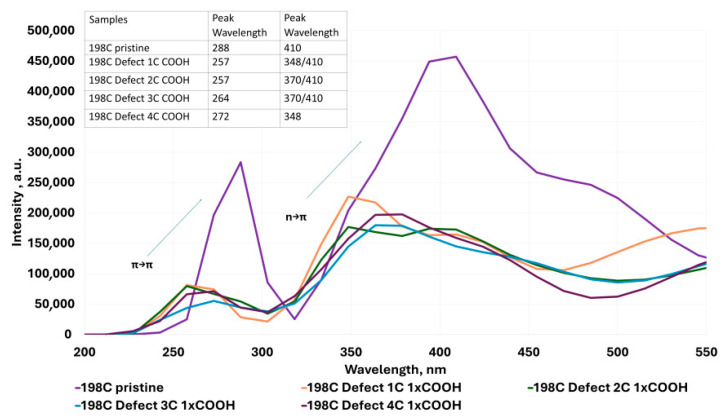
Examples of UV–Vis absorption spectra for the ideal modeled graphene quantum dot system and for defected systems with 1 C atom and -COOH group substituted in the defect region.

**Figure 7:**
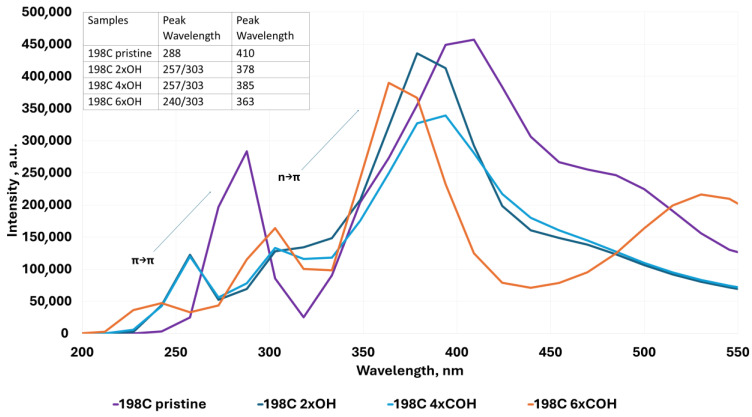
Examples of UV–Vis absorption spectra for the ideal modeled graphene quantum dot system and for layouts with -OH groups 1 to 6 substituted at the edges.

**Figure 8:**
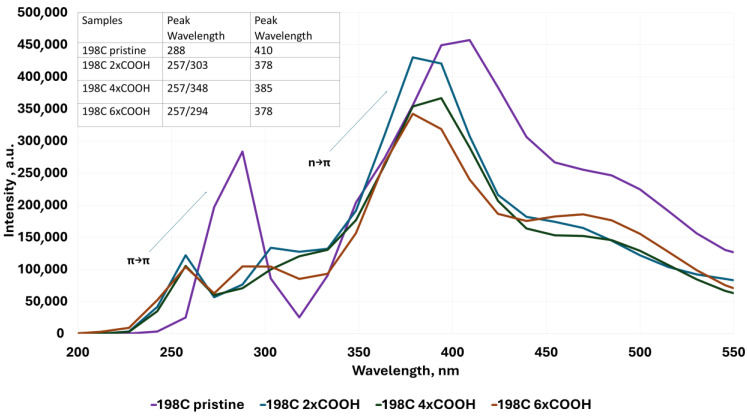
Examples of UV–Vis absorption spectra for the ideal modeled graphene quantum dot system and for layouts with -COOH groups of 1 to 6 substituted at the edges.

**Figure 9:**
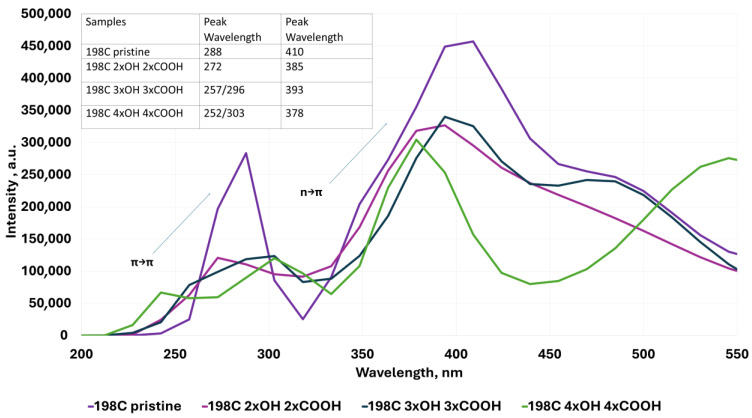
Examples of UV–Vis absorption spectra for the ideal modeled graphene quantum dot system and for layouts with -OH and -COOH groups of 4 to 8 substituted at the edges.

**Figure 10:**
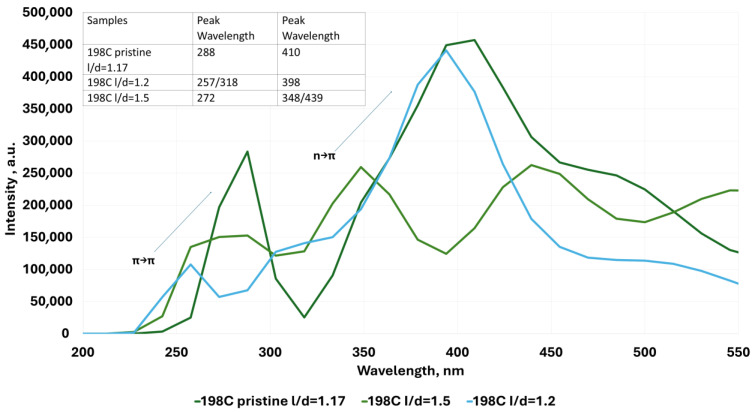
Examples of UV–Vis absorption spectra for the ideal modeled graphene quantum dot system and for layouts with variable l/d parameter.

The UV–Vis absorption spectra shown in Figure 4, Figure 5, Figure 6, Figure 7, Figure 8, Figure 9 and Figure 10 are based on vertical electronic transitions calculated at the semi-empirical CI/PM3 level. The intensity of each peak reflects the oscillator strength associated with the electronic transition and allows qualitative comparison of spectral changes due to structural variation or functionalization.

The identified transitions correspond primarily to π-π* (around 280–300 nm) and *n*-π* (around 370–410 nm) excitations, consistent with previously reported experimental studies of QGDs with similar topology and composition [1,11]. This validates the use of semi-empirical methods for trend analysis, despite known limitations in absolute accuracy.

To determine the influence of the different structures of the modeled QGDs (defects in the form of vacancies, substitution by -OH and -COOH groups, as well as a change in dot dimensions) with their resulting electron properties, UV–Vis absorption spectra were modeled. Analysis of the modeled UV–Vis absorption spectra proves the following:

1. For QGD structures without vacancy defect (Figure 4), two strong absorption peaks are observed at 288 nm and 410 nm, respectively. These peaks are associated with the following [1,2,3]:
  (a) π-π* transition of aromatic molecular of sp^2^ in case of absorption ~280 nm,
  (b) *n*-π* transition for absorption at a wavelength of ~380 nm.
2. Regarding QGD systems with vacancies in the form of C atoms (Figure 4), the following statement can be concluded:
  (a) The presence of structural defects in the range of one to four C atoms moves the position of the peak corresponding to the π-π* transition to lower wavelengths, i.e., from 288 nm for QGD pristine to 264 nm for QGD with a defect of the size of four carbon atoms.
  (b) The presence of structural defects in the range of one to four C atoms displaces the position of the peak relevant to π-π* transition towards lower wavelengths, i.e., from 288 nm for QGD pristine to 264 nm for QGD with a defect size of four carbon atoms.
  (c) A similar relation is observed for the peak corresponding to the *n*-π* transition. In this case, the peak displacement is from a value of 410 nm to 390 nm for a QGD with a defect size of four C atoms.
3. In QGD systems with C atom vacancies and with a substitution in the defect region:
  (a) With one -OH group, it can be observed that the peak corresponding to the π-π* transition moves towards lower wavelength values. The peak representing the *n*-π* transition behaves similarly, shifting towards the UV range for defect sizes of one and two carbon atoms. In contrast, for higher defect values (3 and 4 C atoms), an increase towards the blue color is observed (Figure 5).
  (b) With one -COOH group, a similar trend to the -OH substitution of QGD is observed; however, the peak heights corresponding to the π-π* transition are more than twice as low as the *n*-π* transition (Figure 6).

4. In the case of edge-substituted QGD systems, we found the following:
  (a) With the -OH groups (Figure 7), a shift towards lower wavelength values and the appearance of two peaks corresponding to the π-π* transition in the ~257 nm—π-π* transition in aromatic systems and the ~300 nm—π-π* transition in C=O systems [2], respectively, can be distinctly seen.
  (b) With -COOH groups (Figure 8), a similar relation as for substitution with -OH groups is observed, except that the effect is less marked.
  (c) The simultaneous substitution with -OH and -COOH groups (Figure 9) results in a displacement of the peak towards higher wavelength values corresponding to the π-π* transition, a similar dependence with respect to the selective substitution of QGD only with -COOH groups. In contrast, the peak corresponding to the *n*-π* transition moves slightly towards lower wavelength values.

With regard to QGD systems with a variable form factor l/d (Figure 10), it is seen that an increase in this parameter causes a shift of the peak responsible for the *n*-π* transition towards lower wavelength values. At the same time, a slight offset in the same direction of the peak corresponding to the π-π* transition is observed.

### 2.3. Value Variation of the HOMO–LUMO Gap as a Function of the QGD Defect Type

**Figure 11:**
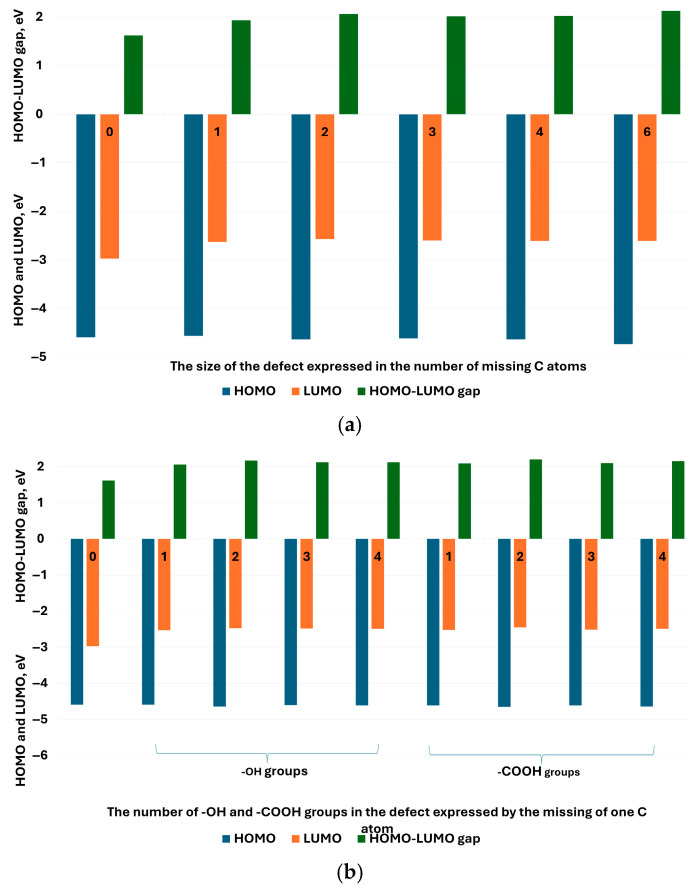

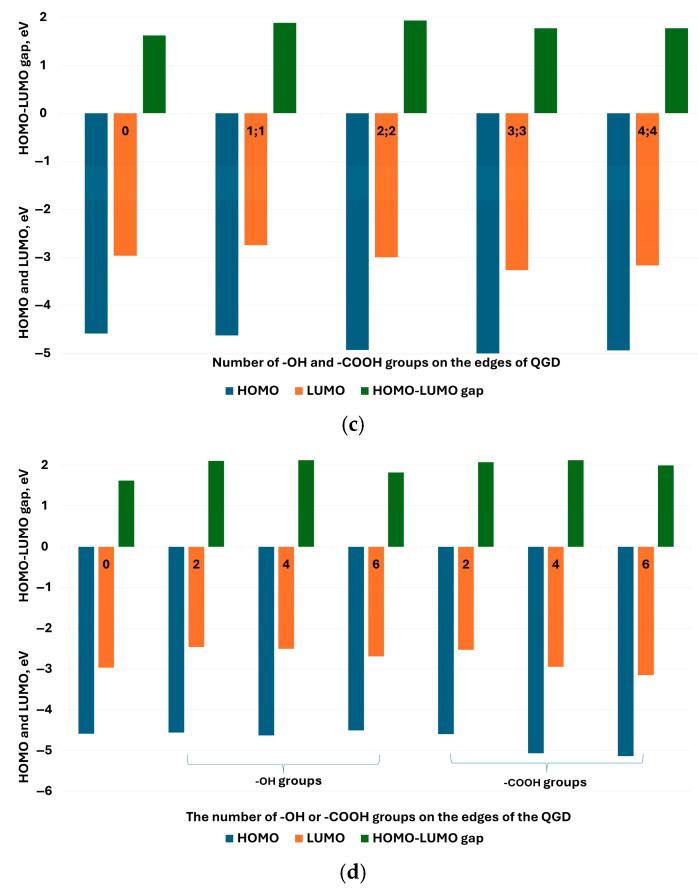
HOMO LUMO values for the modeled QGD systems, respectively: (**a**) in relation to the size of the defect carbon atom vacancy: from 0 to 6 C atoms; (**b**) as a function of the number of -OH or -COOH groups located in the defect—carbon atom vacancy; (**c**) as determined by the simultaneous number of -OH and -COOH groups located at the edges; (**d**) according to the number of -OH or -COOH groups located at the edges.

**Figure 12:**
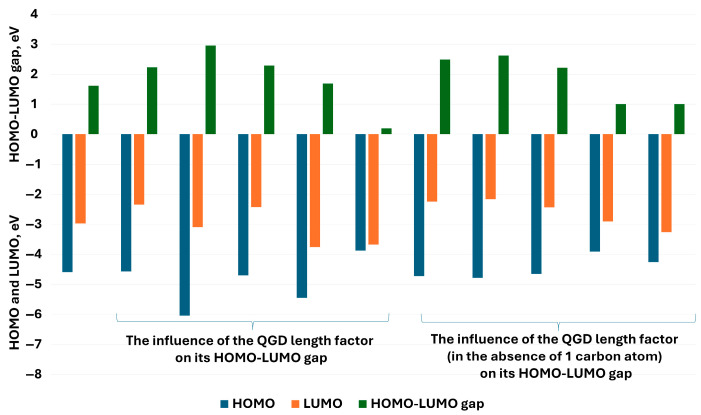
HOMO LUMO values for the modeled QGD systems, respectively: depending on the value of the l/d parameter and according to the value of the l/d parameter with an additional carbon atom vacancy.

Even though the substitution of QGD with -OH or -COOH groups, or with -OH and -COOH, does not change the semiconducting character of the modeled materials, the value of the HOMO–LUMO difference increases towards the insulator-like material. For an undefective QGD structure, the HOMO–LUMO value is 1.62 eV (Figure 11a), this substitution with one -OH or -COOH group increases this value to 2.1 eV and 2.07 eV, respectively (Figure 11b). In the case of the unlikely state (6 -OH or -COOH groups for a QGD system built from 170 carbon atoms), due to the relatively high energy of the system, the value of the HOMO–LUMO difference is 1.82 eV and 1.99 eV, respectively (Figure 11d). A certain exception is the simultaneous substitution with -OH and -COOH groups. In this case, the value of the HOMO–LUMO difference is smaller compared to the same amount of one type of -OH or -COOH groups (Figure 11d). Independently, as the degree of defectivity rises, the value of the LUMO level increases, with a value of about −3 eV for the undefective structure. The HOMO values for all analyzed structures are practically at the identical level around 4.5 eV. Discrepancies appear in the case of QGD analysis depending on the value of the l/d parameter (Figure 12). In this case, the HOMO–LUMO gap reaches a maximum value (approximately 3 eV) for systems for which the value of the l/d ratio is around 2. However, the additional structure defect—the carbon atom vacancy results in the maximum value of the l/d ratio shifting towards its lower values. In the modeled range for the defected QGD HOMO–LUMO system, the gap is about 2.8 eV for an l/d value of 1.5.

### 2.4. Comparison with the Literature

The theoretical results presented here are consistent with numerous experimental and computational reports on QGDs. For instance, Yin et al. [11] report dual emission (green/red) from QGDs with size and surface functionalization as key variables. Nguyen and Kim [1] observed blue-shifted UV absorption with decreasing dot size and increasing defectivity, in agreement with our data in Figure 4 and Figure 5.

Furthermore, Ahmed et al. [9] demonstrated that oxygen-rich functionalization (-OH, -COOH) increases the HOMO–LUMO gap and introduces polarity, consistent with our calculations (Figure 11). Huang et al. [18] emphasized the tunability of electronic transitions in bottom–up synthesized QGDs, reinforcing the role of structural control in optoelectronic behavior.

The trends observed in our simulation data agree with several experimental studies on graphene quantum dots (QGDs) of varying size and defect content. Sun et al. [23] and Peng et al. [24] observed blue-shifting of absorption peaks and increasing HOMO–LUMO gaps in smaller or more defective QGDs, consistent with our calculated shifts from 410 nm to 390 nm and gap changes from 1.6 eV to 3.0 eV. Zhao et al. [25] and Li et al. [26] reported that oxidation and edge functionalization, e.g., -COOH, increase polarity and reduce photoluminescence efficiency due to increased localization of electronic states, also reflected in our electron density maps.

From a physical standpoint, vacancy defects interrupt π-conjugation pathways and localize frontier orbitals, leading to higher HOMO–LUMO gaps. Functional groups such as -OH and -COOH withdraw electron density and stabilize the LUMO more than the HOMO, shifting the absorption towards the UV region. Additionally, increasing the l/d aspect ratio of the QGDs leads to effective quantum confinement along one dimension, increasing excitation energy and reducing delocalization, as observed in Figure 12. This is in line with theoretical predictions by Ahmed et al. [9] and experimental validations by Desmond et al. [27].

Overall, our work confirms that structural defects, edge composition, and geometry strongly influence the optoelectronic response of QGDs, which must be considered during synthesis design.

## 3. Methodology

To verify the physicochemical properties of graphene quantum dots (QGD) in conjunction with the structure, based on quantum theory, chemical calculations were performed using the semi-empirical MO-G PM3 method implemented in Fujitsu Scigress. Molecular geometries were fully optimized in vacuum with convergence criteria set to a gradient norm of 0.05 kcal/mol/Å.

We used configuration interaction (CI), including up to 10 excited states, for excited-state analysis and UV–Vis absorption spectra. Oscillator strengths (f-values) were calculated and used to construct absorption spectra plotted in arbitrary units (a.u.) and normalized per system.

No solvent model was applied, as the goal was to evaluate the intrinsic electronic effects caused by structural variation and defects. The systems contained up to ~200 atoms (C, H, O), and no periodic boundary conditions were employed.

1. Changes in UV–Vis absorption spectra depending on the type of QGD defect were determined.
  (a) In this case, structural defects in the form of vacancies of carbon atoms 1 to 6 were modeled.
2. Changes in the HOMO–LUMO gap and UV–Vis absorption spectra were determined as a dependence on the following:
  (a) On the type and number of -COOH; -OH functional groups located at the edges of the analyzed structures.
  (b) The type and number of -COOH; -OH functional groups placed in structural defects.
  (c) Rectangle geometry was chosen to model QGD with parameter l/d where l is the length of the quantum dot and d is its width parameter.
3. For the QGD structures modeled as described in this paper, analyses of the electron distribution on their surfaces were conducted in order to illustrate also the possibility of the formation of dipole systems on their surfaces, which may also be relevant in the context of their interaction with each other.

The analyses were undertaken for conditions in which the QGDs were isolated from the environment (no additional external interactions). Due to the presence of only three types of atoms (O, H and C), a semi-empirical method was used, which enabled relatively fast calculations. The analyzed QGD systems, depending on the type of defect, were constructed with a total of about 200 carbon, hydrogen and oxygen atoms.

UV–Vis spectra were simulated using the configuration interaction (CI) approach based on the MO-G PM3 semi-empirical method implemented in Fujitsu Scigress. MO-G PM3 is a modified variant of the Parametric Method 3 (PM3), adapted to graph-based molecular orbital representations, which enables simulations of large carbon-based systems. The 10 lowest singlet excited states were computed for each optimized QGD structure. Oscillator strengths (f-values), derived from transition dipole moments, were extracted and used to generate stick spectra. No solvent model was applied; all calculations were performed in vacuum to focus on the intrinsic quantum effects of structure and defects. Spectra were plotted in arbitrary units and normalized per system using Gaussian convolution.

## 4. Conclusions

1. The molecular modeling performed confirmed that the same values of HOMO–LUMO gap for different QGDs can imply the same color emissions in fluorescence. Therefore, different structures and thus systems with different physicochemical properties can give the same responses in fluorescence. This does not mean that they exhibit the same structural characteristics. This fact may translate into a variable magnitude of energy emission when an electron returns from the excited state.
2. It is apparent that the value of the HOMO–LUMO gap grows with increasing defectivity, which is directly related to the decreasing degree of coupling in the QGD structure.
3. Irrespective of the size of the QGDs, their defects in the form of carbon atom vacancies, substitution with -OH and/or -COOH causes a change in electron excitation energy and consequently a change in color. Furthermore, there is a change in the electron distribution causing QGDs substituted with -OH and/or -COOH to exhibit a significantly polar character due to an increase in the proportion of O atoms, which shows a strong electronegative character.
4. An identical relationship is observed for the shape change in the QGD, where the elongation of its structure changes the excitation range of the electron. Then the transitions of the valence electrons to a higher energy level occur for lower values of the radiation wavelength.

## Data Availability

The original contributions presented in this study are included in the article. Further inquiries can be directed to the corresponding author.

## References

[1] Nguyen D.K., Kim T.Y. (2018). Graphene quantum dots produced by exfoliation of intercalated graphite nanoparticles and their application for temperature sensors. Appl. Surf. Sci..

[2] Huang H., Yang S., Li Q., Yang Y., Wang G., You X., Mao B., Wang H., Ma Y., He P. (2018). Electrochemical Cutting in Weak Aqueous Electrolytes: The Strategy for Efficient and Controllable Preparation of Graphene Quantum Dots. Langmuir ACS J. Surf. Colloids.

[3] Ahirwar S., Mallick S., Bahadur D. (2017). Electrochemical Method to Prepare Graphene Quantum Dots and Graphene Oxide Quantum Dots. ACS Omega.

[4] Islam M.S., Deng Y., Tong L., Roy A.K., Faisal S.N., Hassan M., Minett A.I., Gomes V.G. (2017). In-situ direct grafting of graphene quantum dots onto carbon fibre by low temperature chemical synthesis for high performance flexible fabric supercapacitor. Mater. Today Commun..

[5] Dong Y., Pang H., Ren S., Chen C., Chi Y., Yu T. (2013). Etching single-wall carbon nanotubes into green and yellow single-layer graphene quantum dots. Scopus.

[6] Zhao Y., Wu X., Sun S., Ma L., Zhang L., Lin H. (2017). A facile and high-efficient approach to yellow emissive graphene quantum dots from graphene oxide. Carbon.

[7] Bruno F., Sciortino A., Buscarino G., Soriano M.L., Rios A., Cannas M., Gelardi F., Messina F., Agnello S. (2021). A Comparative Study of Top-Down and Bottom-Up Carbon Nanodots and Their Interaction with Mercury Ions. Nanomaterials.

[8] Cui Y., Liu L., Shi M., Wang Y., Meng X., Chen Y., Huang Q., Liu C. (2024). A Review of Advances in Graphene Quantum Dots: From Preparation and Modification Methods to Application. C.

[9] Ahmed D.S., Mohammed M.K.A., Majeed S.M. (2020). Green Synthesis of Eco-Friendly Graphene Quantum Dots for Highly Efficient Perovskite Solar Cells. ACS Appl. Energy Mater..

[10] Xu Q., Niu Y., Li J., Yang Z., Gao J., Ding L., Ni H., Zhu P., Liu Y., Tang Y. (2022). Recent progress of quantum dots for energy storage applications. Carbon Neutrality.

[11] Yin L., Zhang D., Li W., Hu Y., Wang L., Zhang J. (2022). White light emitting diodes based on green graphene quantum dots and red graphene quantum dots. Mol. Cryst. Liq. Cryst..

[12] Ramachandran P., Khor B.K., Lee C.Y., Doong R.A., Oon C.E., Thanh N.T., Lee H.L. (2022). N-Doped Graphene Quantum Dots/Titanium Dioxide Nanocomposites: A Study of ROS-Forming Mechanisms, Cytotoxicity and Photodynamic Therapy. Biomedicines.

[13] Kadian S., Sethi S.K., Manik G. (2021). Recent advancements in synthesis and property control of graphene quantum dots for biomedical and optoelectronic applications. Mater. Chem. Front..

[14] Zheng B., Chen Y., Li P., Wang Z., Cao B., Qi F., Liu J., Qiu Z., Zhang W. (2017). Ultrafast ammonia-driven, microwave-assisted synthesis of nitrogen-doped graphene quantum dots and their optical properties. Nanophotonics.

[15] Shehab M., Ebrahim S., Soliman M. (2017). Graphene quantum dots prepared from glucose as optical sensor for glucose. J. Lumin..

[16] Kumar P.S., Aziz S.K.T., Girshevitz O., Nessim G.D. (2018). One-Step Synthesis of N-Doped Graphene Quantum Dots from Chitosan as a Sole Precursor Using Chemical Vapor Deposition. J. Chem. C.

[17] Deka M.J., Chowdhury D. (2017). CVD Assisted Hydrophobic Graphene Quantum Dots: Fluorescence Sensor for Aromatic Amino Acids. Chem. Sel..

[18] Huang D., Zhou H., Wu Y., Wang T., Sun L., Gao P., Sun Y., Huang H., Zhou G., Hu J. (2019). Bottom-up synthesis and structural design strategy for graphene quantum dots with tunable emission to near infrared region. Carbon.

[19] Singh R.K., Kumar R., Singh D.P., Savu R., Moshkalev S.A. (2019). Progress in microwave assisted synthesis of quantum dots (graphene/carbon/semiconducting) for bioapplications: A review. Mater. Today Chem..

[20] Zhao C., Song X., Liu Y., Fu Y., Ye L., Wang N., Wang F., Li L., Mohammadniaei M., Zhang M. (2020). Synthesis of graphene quantum dots and their applications in drug delivery. J. Nanobiotechnol..

[21] Niu C., Yao Z., Jiang S. (2023). Synthesis and application of quantum dots in detection of environmental contaminants in food: A comprehensive review. Sci. Total Environ..

[22] Sabetghadam S.A., Hosseini Z., Zarei S., Ghanbari T. (2020). Improvement of the current generation in silicon solar cells by utilizing graphene quantum dot as spectral converter. Mater. Lett..

[23] Sun X., Liu Z., Welsher K., Robinson J.T., Goodwin A., Zaric S., Dai H. (2008). Nano-graphene oxide for cellular imaging and drug delivery. Nano Res..

[24] Peng J., Gao W., Gupta B.K., Liu Z., Romero-Aburto R., Ge L., Song L., Alemany L.B., Zhan X., Gao G. (2012). Graphene quantum dots derived from carbon fibers. Nano Lett..

[25] Zhao A., Chen Z., Zhao C., Gao N., Ren J., Qu X. (2020). Recent advances in bioapplications of C-dots. Carbon.

[26] Li X., Yu L., He M., Chen C., Yu Z., Jiang S., Wang Y., Li L., Li B., Wang G. (2023). Review on carbon dots: Synthesis and application in biology field. BMEMat.

[27] Desmond L.J., Phan A.N., Gentile P. (2021). Critical overview on the green synthesis of carbon quantum dots and their application for cancer therapy. Environ. Sci. Nano.

